# Challenges for first contact physiotherapists' managing sickness absence: Consensus development using the nominal group technique

**DOI:** 10.1177/02692155241300089

**Published:** 2024-12-20

**Authors:** Cameron Black, Sivaramkumar Shanmugam, Heather Gray

**Affiliations:** 1 Buckinghamshire Healthcare NHS Trust, Aylesbury, UK; 2 Department of Physiotherapy and Paramedicine, Glasgow Caledonian University, Glasgow, Scotland; 3 15564 Healthcare Improvement Scotland, Glasgow, UK

**Keywords:** sickness absence, health care, primary care, physiotherapists

## Abstract

**Objective:**

To identify the challenges and key learning and development needs of First Contact Physiotherapists (FCPs) providing fitness for work and sickness absence certification from Occupational Health physiotherpists' viewpoints.

**Design:**

An online modified version of the Nominal Group Technique.

**Participants:**

A convenience sample of 21 expert occupational health physiotherapists as participants whose substantive job role was within a public or private UK based occupational health provider.

**Main Measure:**

Consensus on each competency was defined a priori as an agreement of more than 60%.

**Results:**

Nine items ultimately reached the required 60% threshold consensus level from the Occupational Health expert group for Question 1 on the challenges involved in providing fitness for work and sickness absence certification. Of these items, five reached full consensus; two of which (‘Time’ and ‘Lack of knowledge’) were deemed the most important items. For Question 2 on the learning and development needs, six items reached full consensus from the group (100% of participants that ranked an item) and two of these items (‘Work conversations’ and ‘Training in Occupational Health topics’) both reached full consensus from the group and were deemed the most important items.

**Conclusion:**

Most employees in the UK do not have access to Occupational Health services. Fit Notes can be an important vehicle to provide work-related, fitness for work and sickness absence advice to help prevent long-term sickness absence. This study provides insights into some barriers and educational development needs of FCPs in primary care, as judged by an expert Occupational Health physiotherapy group.

## Introduction

First Contact Physiotherapists (FCPs) improve therapeutic outcomes in primary care^1^ and with further training and development, the expectation of improved work-related outcomes.^2^ Defining their required health and work competencies is essential for delivering consistent, evidence-based advice. One new but important area for FCPs involves Fit Notes, though this tool remains underutilised. Originally designed to facilitate timely return to work, Fit Notes have become predominantly used as absence certificates. In 2020, 94% of Fit Notes issued by General Practitioners lacked workplace adjustment recommendations.^3^ This is at odds with their original purpose of enabling clinicians to provide work fitness guidance. By 2016, the UK Government acknowledged that Fit Notes were ‘not fully achieving’ their intended purpose.^4^

July 2022 saw UK expanded Fit Note certification to include FCPs. This expansion is crucial as only 50% of UK employees have access to Occupational Health services.^5^ This was first documented in the UK's Departments of Health and Work and Pensions White Paper *Improving Lives: The Future of Work, Health and Disability.*^6^ The Fit Note was introduced in the UK in 2010 based on Dame Carol Black's review^7^ and it attempted to help keep individuals in work while living through a period of mental or physical ill health, to the advantage of the employee, employer, and society overall. Despite these developments, Fit Notes primarily function as ‘Sick Notes’, missing opportunities to provide work adjustment guidance.^7,8^

This study aims to identify the challenges and key learning and development needs for FCPs to provide work-specific advice in primary care settings. While FCPs are increasingly involved in fitness-for-work assessments, this represents a new responsibility, and guidance regarding necessary competencies remains limited. To address this gap, this study assembled an expert panel of Occupational Health physiotherapists to identify essential work-related competencies for FCPs in managing Fit Notes and conducting effective work-health discussions. Although Occupational Health physiotherapists may not work as FCPs, their deep expertise in occupational health makes them uniquely qualified to identify the competencies and skills required to integrate work-related advice into clinical practice. Their input provides an important foundation for the development of FCP's capabilities in this area, complementing the practical insights of FCPs in future research. This two-step approach ensures that we first establish a strong competency framework, which can later be validated and refined by FCPs in the context of primary care.

Previous studies,^2,9^ examined challenges but did not specify required competencies. This contrasts with clinical frameworks advocating for regular work discussions and use of Fit Notes. Therefore, this study aims to move forward by defining the specific competencies required for FCPs to fulfil their evolving role in primary care effectively.

## Methods

### Design

A novel online modification of the Nominal Group Technique was used.^2^ Physiotherapists like FCPs regularly make challenging choices about treatment options in the face of undiagnosed presentations and an uncertain clinical picture. In research settings, consensus group methods can be used to determine components of a new or revised curriculum, define competencies and develop learning and educational resources.^10^ They are used as a systematic means for developing, reaching, and measuring consensus.^11,12^ The Nominal Group Technique was developed as a method that established a structured, in-person group meeting with common hallmarks of: silent idea generation, round robin interaction, clarification and ranking.^13^ The face-to-face interaction usually involves 5–12 participants and can run from 1.5 to 6 hours.^13^ The Nominal Group Technique is a well-established idea generation tool, especially when stakeholders are faced with conditions of uncertainty or incomplete evidence, in health, economics, education and industry.^7^ Advantages of this method include the generation of a larger number and quality of ideas, the potential for discussion and debate and unique interpretive and consensual data.^14–16^ It can also support the democratic representation of wide ranging opinion with equal participation as long as effectively managed by researchers.^12^

### Participant recruitment and data collection process

Participants provided written informed consent to be included in the study. Recruitment started when ethical approval was granted on 28th February 2020 and concluded on the 14th April 2020. Ethical approval was granted by Glasgow Caledonian University's Health and Life Sciences Research Ethics Committee (Reference: HLS/PSWAHS/19/144).

Data were gathered from experts defined as Occupational Health physiotherapists involved in management of musculoskeletal conditions in both public and private sectors, providing fitness for work advice and sickness absence information. These specialists were selected for their considerable knowledge and skills in Occupational Health, having a shared commitment to improving the health and wellbeing of workers. Most participants were members of a professional network that has published a competency framework for physiotherapists working in Occupational Health. This framework promotes the development needs of Allied Health Professions and outlines the necessary knowledge, skills, and behaviours for delivering quality care.^17^

Given the focus on FCPs handling work and health tasks, recruiting these experts was crucial for obtaining insights relevant to the field. Their experiences reflect the challenges faced in primary care, where patients often seek discussions about work-related issues but frequently miss the opportunity to engage with their healthcare providers. Through this expert panel, the study aims to facilitate the ongoing development and refinement of existing competency frameworks and educational programs.

We aimed to recruit 20–25 participants, anticipating this number would provide a balance between a diverse range of opinions and the practicalities of facilitating online group discussions. Recruitment was conducted through purposive and snowball sampling via professional networks such as the Chartered Society of Physiotherapy and the Association of Chartered Physiotherapists in Occupational Health and Ergonomics, as well as social media platforms like X (previously Twitter) (@black_cameron).^18^ Twenty-one participants were successfully recruited across two groups. Efforts were made to ensure representation from both public and private sectors, recognising the importance of diverse perspectives in shaping consensus. Participants were selected based on their involvement in managing musculoskeletal conditions in occupational health settings, ensuring relevant expertise for the study's focus. Some participants assisted in recruitment via snowballing^13^ by sharing within their local Association of Chartered Physiotherapists in Occupational Health and Ergonomics networks and via social media.

To accommodate to the online modified Nominal Group Technique, it was decided to run two nominal groups due to the number of Occupational Health experts, professional re-deployments and because some clinicians were needed professionally to meet the COVID-19 response. Groups were formed based on availability, but efforts were made to keep group sizes similar. This convenience-based approach was due to the unique challenges posed by COVID-19, but we recognise that this could have introduced variability into the group dynamics. We aimed to minimise this impact by ensuring consistency in facilitation and process across both groups. The potential variability in group sizes was acknowledged as a limitation that could affect group dynamics and the final consensus. However, the Nominal Group Technique process, which involves independent ranking by participants, helps mitigate this by ensuring that individual input is still accounted for in the overall results, regardless of group size.

**Figure 1.**
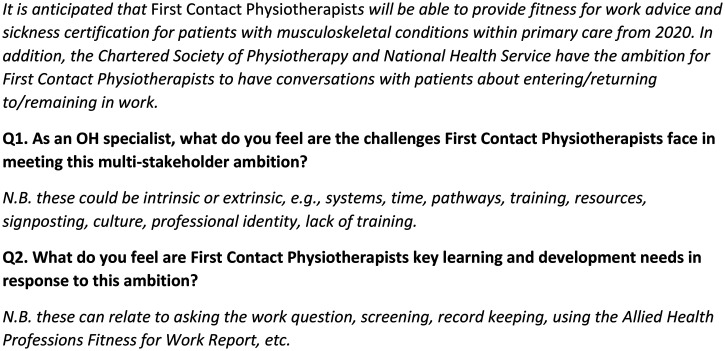
Questions considered during online Nominal Group Technique meetings.

Participants were provided with an information sheet, informed consent form, and completed a Doodle^TM^ poll to confirm when they could attend the online nominal group. An online digital pre-session information guide and instructions were provided one week before via email, including information on the Nominal Group Technique questions, demographic data questionnaire, a welcome statement and information on meeting joining instructions. Blackboard Collaborate^TM^ was the real-time video conferencing tool used to conduct the online meeting. From the pre-session email, participants started the Nominal Group Technique first step of idea generation by sharing content on their initial ideas on the topic via a digital canvas software (Padlet^TM^). The 2-hour structured online nominal groups were conducted involving structured moderator led discussion to identify items, followed by individual ranking of these items, thereby allowing all experts to contribute equally. The meeting focused on problem-solving and idea-generating strategies to answer the two questions (Figure 1).

**Figure 2.**
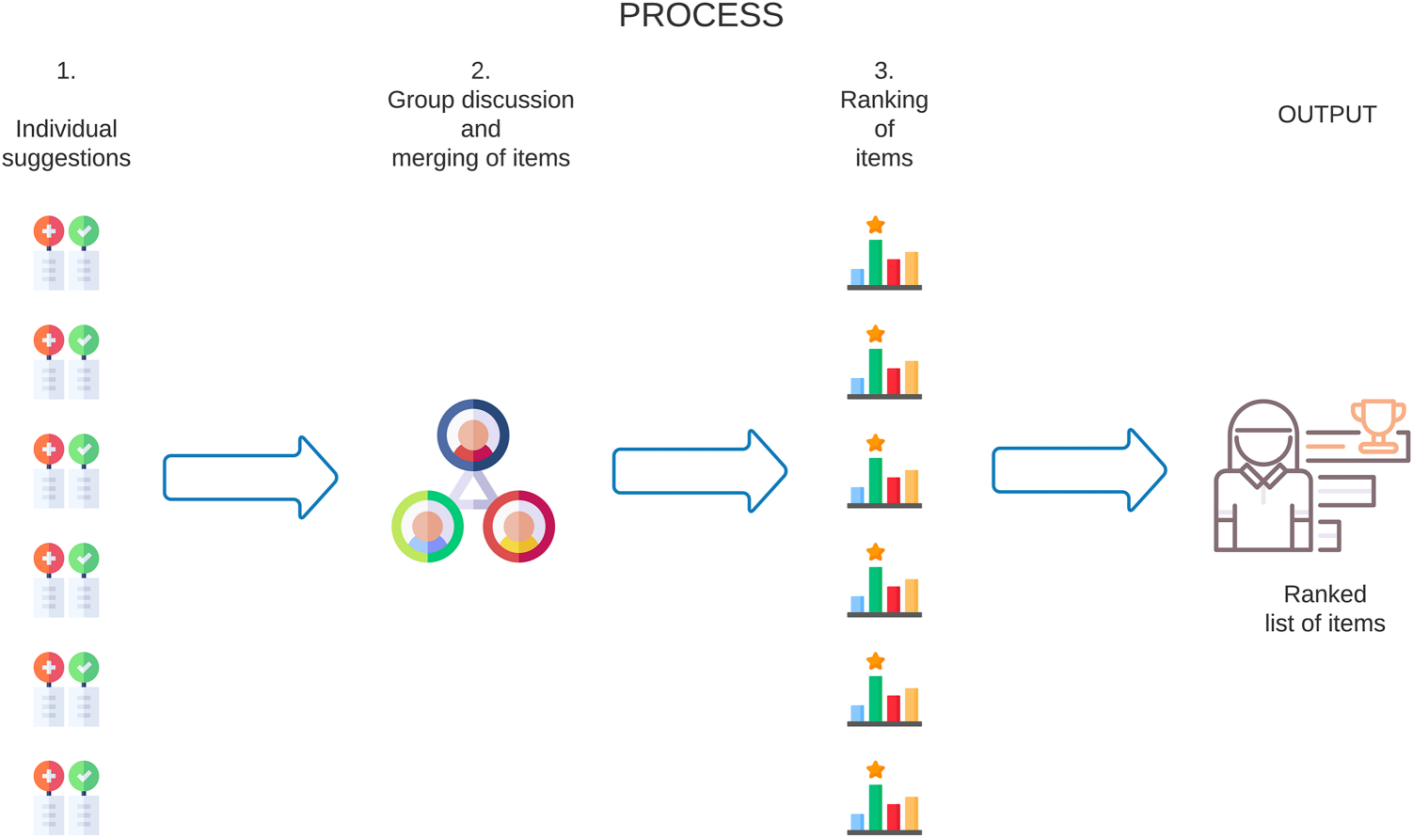
Online Nominal Group Technique process detailing the stages of the meeting.^14^

In this study, Nominal Group Technique was modified for an online setting and streamlined into three phases (Figure 2): (1) discussion and clarification of the question and items relating to FCP practice from the pre-populated Padlet; (2) individual rankings of each item's importance were conducted using a shared Google Sheet, with all participants utilising the same electronic spreadsheet. Guidance on data sharing and interaction was provided during the session to facilitate this process; and (3) rank items based on their perceived importance or relevance to the specific question, which facilitates the identification of consensus. Participants ranked the five most important items following clear guidance on how the results would be compiled and collated to reflect the group's overall priorities.

This was conducted for both Nominal Group Technique questions. Three researchers (CB, HG and SS) facilitated the groups with the question guide provided in Figure 1. It was anticipated that FCPs would be legislated for sickness absence certification at the time of data, but due to COVID-19 delays, this did not occur. It was decided to keep the same wording in anticipation of future legislation. The Nominal Group Technique sessions were recorded using audio and video through Blackboard Collaborate^TM^ to provide clarification of the items as needed.

To ensure consistency in the results, data from the two groups were amalgamated by combining the rankings from both groups into a single dataset. The individual rankings were compiled in a shared Google Sheet, and the final consensus was reached by calculating the mean reciprocal ranks for each item across both groups, allowing for an integrated analysis of the prioritised items while preserving each group's contributions.

### Data analysis

Descriptive analyses were conducted to present demographic data. The level of consensus (percent of participants that ranked an item) was defined a priori to be at the 60% consensus level in keeping with criterion for accepted consensus levels within other consensus methods in the published literature.^12^ While this is consistent with established practices, it is acknowledged that this lower threshold may limit the strength of agreement. The items generated in the Nominal Group Technique meeting were ranked as mentioned (1 = most important, 5 = least important). Participants could choose not to rank any items that they did not consider relevant to the question. A median rank score and the number of times participants scored a specific item (frequency) were also recorded. The frequency was used to calculate the percentage of experts who ranked an item.^14^

An importance score for each item was computed as the average of the reciprocal rankings, this process is proposed by Cho et al.^15^ and through a similar rationale to the Expected Reciprocal Rank Evaluation proposed in a different context.^16^ An importance score (0–1) was ascertained for each item from its mean reciprocal rank, with scores closer to 1 deemed more important. The mean reciprocal rank is 1 divided by the ranking by each participant – 1 for the item ranked in first place, a half for second place, one-third for third place, and so on. For example, if *communication* was ranked first by one participant and fourth by another, the reciprocal rankings would be 1 and one-fourth, respectively. If an item was not ranked, it was assigned a zero. The importance given to an outcome was thus measured both by its ranking and by the consistency of it being nominated by the participants. The reason for inverting the ranks is to give more weight to top ranks and less to the lower ranks. Higher values of the score identify outcomes that are more valued by the participants. For example, if all group participants ranked a specific item 1 respectively, this would mean 100% consensus and an importance score of 1. In other words, the group not only fully agreed on the item's inclusion in the final consensus, but also ranked it as the most important item. It is suggested that items should be assessed in terms of the score as well as the frequency of votes, as this may be more representative of overall group priority of items.^14^

## Results

In total, 21 experts (average (mean) age: 44 years, age range 29–58 years, 14 were female and seven male) participated across the two nominal groups with an average (mean) of 22 years’ professional physiotherapy practice. Most participants were registered members of the Association of Chartered Physiotherapists in Occupational Health and Ergonomics (90% of the total participants in the group, the remainder worked in National Health Service or private Occupational Health providers but were not registered with the Association of Chartered Physiotherapists in Occupational Health and Ergonomics (10%)). All had been working in an Occupational Health role for on average (mean) of 15.5 years (years range 5–26). None of the experts worked within an FCP physiotherapy role.

**Figure 3.**
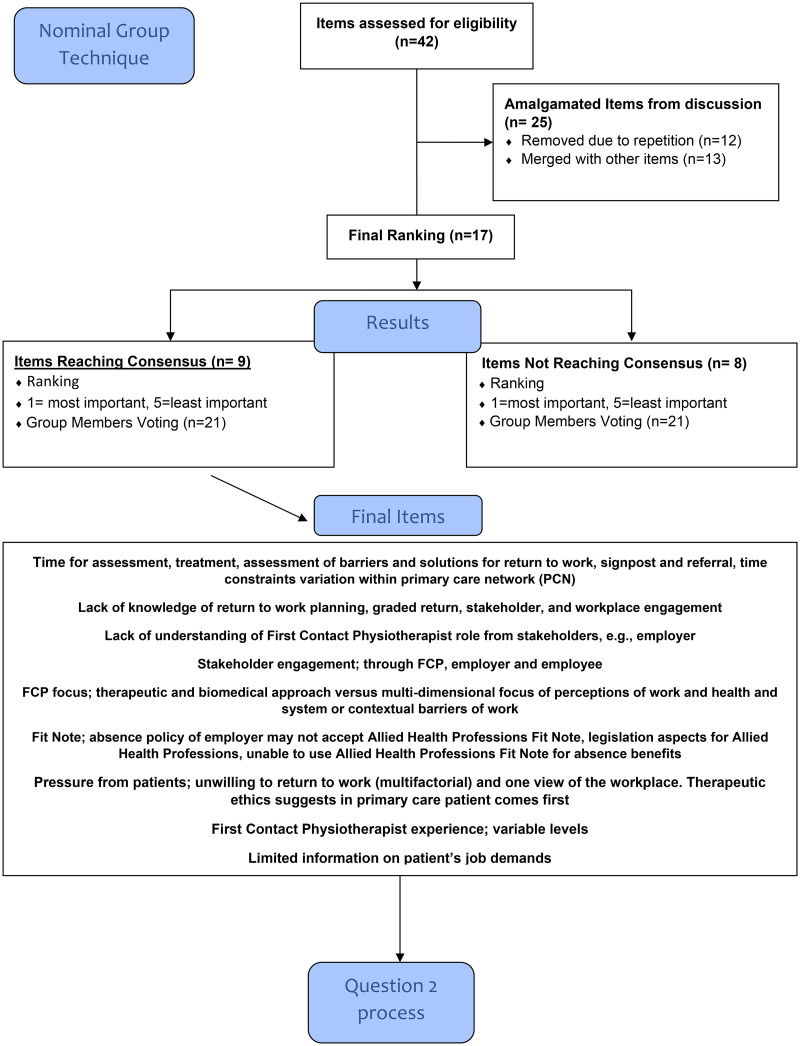
Nominal Group Technique Question 1.

A total of 42 items were generated in response to the first question regarding the (perceived) challenges faced when working as an FCP and providing fitness for work advice, sickness absence certification and work-related conversations (Figure 3). These challenges included: the lack of knowledge of Occupational Health topics and understanding needed to have focussed conversations; intrinsic factors such as the FCP therapeutic role and extrinsic factors such as time; stakeholder understanding of FCP roles and pressure from patients. Nine items ultimately reached the required 60% threshold consensus level (percent of participants that ranked an item) from the Occupational Health expert group (Table 1): ‘Time’, ‘Lack of knowledge’, ‘Lack of understanding’, ‘Stakeholder engagement’, ‘FCP focus’, ‘Fit Note’, ‘Pressure from patients’, ‘FCP experience’ and ‘Limited information on patient's job demands.’ Of these items, five reached full consensus and two items (‘Time’ and ‘Lack of knowledge’) reached full consensus and were deemed the most important items.

**Table 1.** Expert-defined items that reached consensus for question 1.

Items for question 1	Consensus level (% of participants)	Importance score (mean reciprocal rank)	Median rank
*Time* for assessment, treatment, assessment of barriers and solutions for RTW, signpost and referral, time constraints variation within primary care network	100^b^	0.7^a^	1
*Lack of knowledge* of RTW planning, graded return, stakeholder and workplace engagement	100^b^	0.7^a^	1
*Lack of understanding* of FCP role from stakeholders, for example employer	100^b^	0.6	2
*Stakeholder engagement*; through FCP, employer and employee	100^b^	0.4	3
*FCP focus*; therapeutic and biomedical approach versus multi-dimensional focus of perceptions of work and health and system or contextual barriers of work	100^b^	0.4	3
*Fit Note*; absence policy of employer may not accept AHP Fit Note, legislation aspects for AHPs, unable to use AHP Fit Note for absence benefits	78	0.3	4
*Pressure from patients*; unwilling to RTW (multifactorial) and one view of the workplace. Therapeutic ethics suggests in primary care patient comes first	78	0.4	3
*FCP experience*; variable levels	67	0.2	4.5
*Limited information on patient's job demands*	67	0.2	4

FCP: First Contact Physiotherapist; RTW: return to work ability; AHP: allied health professions.

Eight items did not reach consensus for Q1: Limited time and resources to provide Occupational Health advice and communication to stakeholders, Unrealistic expectations on FCP (patient/employee, employer and process), to assess Occupational Health-specific information, Conflict with Occupational Health advice, Role conflict, Lack of opportunity to discuss Occupational Health cases with expert, No opportunity for employer relationship building and Employee expectation that Fit Note completed by General Practitioners solely.

a Most important item(s).

b Full consensus on item(s).

**Figure 4.**
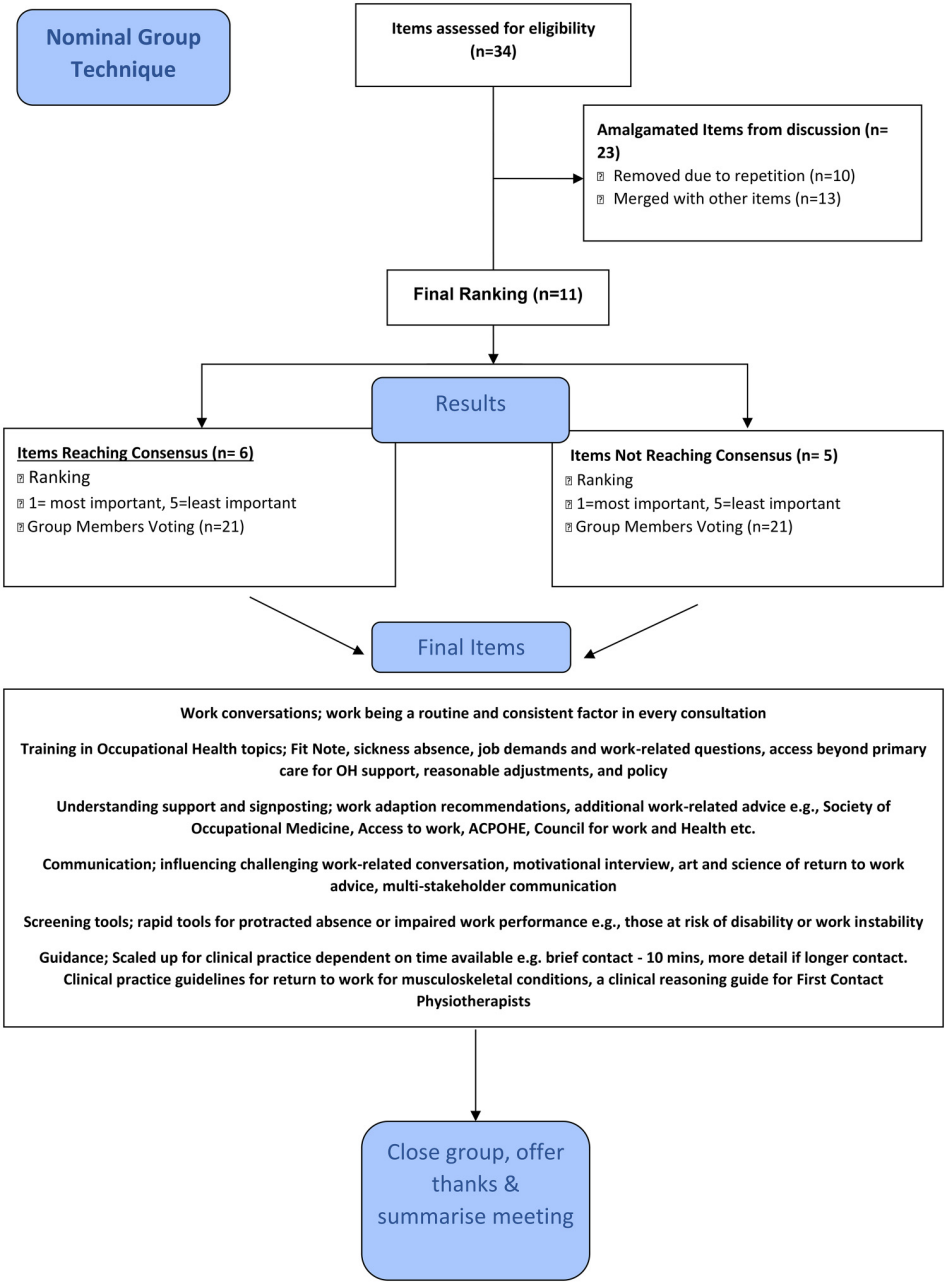
Nominal Group Technique Question 2.

For the second question, 34 items were generated (Figure 4) for the key learning and development needs in relation to the identified ambition with six reaching the required consensus level: ‘Work conversations’, ‘Training in Occupational Health topics’, ‘Understanding support and signposting’, ‘Communication’, ‘Screening tools’ and ‘Guidance’ (Table 2). All six of these items reached full consensus from the group (100% of participants that ranked an item) and two of these items (‘Work conversations’ and ‘Training in Occupational Health topics’) reached full consensus from the group and were deemed the most important items.

**Table 2.** Expert-defined items that reached consensus for Question 2.

Items for question 2	Consensus level (percent of participants)	Importance score (mean reciprocal rank)	Median rank
*Work conversations*; work being a routine and consistent factor in every consultation	100^b^	0.6^a^	2
*Training in Occupational Health topics*; Fit Note, sickness absence, job demands and work-related questions, access beyond primary care for Occupational Health support, reasonable adjustments, and policy	100^b^	0.6^a^	2
*Understanding support and signposting*; work adaption recommendations, additional work-related advice for example SOM, Access to work, ACPOHE, Council for work and Health, etc.	100^b^	0.4	3
*Communication*; influencing challenging work-related conversation, motivational interview, art and science of RTW advice, multi-stakeholder communication	100^b^	0.4	3
*Screening tools*; rapid tools for protracted absence or impaired work performance for example those at risk of disability or work instability	100^b^	0.4	4
*Guidance*; Scaled up guidance for clinical practice dependent on time available for example brief contact for 10 min, more detail if longer contact. Clinical practice guidelines for RTW for musculoskeletal conditions, a clinical reasoning guide for FCPs	100^b^	0.3	5

FCP: first contact physiotherapist; ACPOHE: Association of Chartered Physiotherapists in Occupational Health and Ergonomic; SOM: Society of Occupational Medicine; RTW: return to work.

Five items did not reach consensus for Q2: Outcome measures for progress review of (Occupational Health-related factors, Work capability assessment, Review of progress made (or regression), Improved collaborative communication processes between MDT, employee/patient and employer and Data access of job demand.

a Most important item(s).

b Full consensus on item(s).

## Discussion

The UK's National Health Service has implemented FCP practice across primary care settings^1^ with support for skill acquisition in this work and health area.^6^ Given that Occupational Health services are generally not included in the National Health Service, primary care often becomes the primary setting where patients discuss work-related concerns, especially with musculoskeletal or mental health issues. Primary care serves as both the ‘front door’ and ‘cornerstone’ of the National Health Service, providing a critical opportunity for early intervention. Yet, addressing work-related needs remains a frequently unmet goal, despite a strategic aim for working age patients to receive work-related advice and support, based on the principle that ‘good work is good for health’.^6^ As the working age demographic shifts, with individuals between ages 50 and the state pension age expected to rise from 26% in 2012 to 34% by 2050, supporting work ability will be essential for workforce sustainability.^18^ This is particularly relevant as 13.7 million working age adults in the UK have long-term conditions, including 8.3 million with disabilities impacting daily life.^19,20^ Helping these individuals remain employed could reduce health disparities and support economic stability.

The extent of sickness absence in the UK highlights the importance of primary care in addressing this issue. In 2019, 138 million working days were lost to sickness absence, with approximately 1.4 million individuals experiencing prolonged absences of four weeks or more.^20^ The COVID-19 pandemic has further affected work ability, with 45% of UK long COVID patients reducing work hours post-infection, and European data showing 60% of employed patients with extended symptoms unable to return to work.^21,22^ Thus, providing evidence informed advice on work participation in primary care is important. If FCP challenges and learning needs are met, they could play an important role in reducing sickness absence and improving productivity, enhancing long-term musculoskeletal health. The National Health Service's Long-Term Plan emphasises prevention, tackling health inequalities, and workforce capacity expansion, suggesting that the FCP model could advance work related health outcomes.^23^

This study offers the first evidence of challenges and learning needs that FCPs encounter when providing fitness for work advice and managing sickness absence in primary care. Using insights from Occupational Health experts with specialised expertise, this study identifies nearly 95% of Fit Notes as still being used as ‘sick notes,’ classifying patients as unfit for work rather than supporting limited work engagement where possible.^24^ As economic inactivity from long-term sickness grows, governments in the UK are considering reforms to encourage sustained employment among individuals with health conditions. Preventing long-term sickness absence from the outset may be essential in achieving this goal.

The Nominal Group Technique facilitated rich, consensus driven data by allowing Occupational Health experts to engage directly on this topic. These experts identified ‘time constraints’ and ‘lack of knowledge’ in return to work planning, graded return procedures, and workplace engagement as key barriers for FCPs. Experts recommend that FCPs follow competencies in the Primary Care Educational Roadmap, developed through structured training or portfolio-based approaches.^25^ This framework aligns with observations that FCPs currently lack training in workplace engagement and structured return to work planning, limiting their capacity for comprehensive support during brief consultations.

For question 2, ‘work conversations’ and ‘training in Occupational Health topics,’ were ranked the most important, with experts stressing consistent dialogue and specific Occupational Health training, possibly through bodies like the Association of Chartered Physiotherapists in Occupational Health and Ergonomics or the Society of Occupational Medicine. Work focused consultations should incorporate questions about work, recovery factors, Fit Note guidance, and return to work planning^24^ as evidence indicates that excluding work focused discussions hinders patients’ workforce participation.^26^ However, healthcare providers often view work discussions as beyond their scope due to time, financial, and procedural constraints.^27–29^

Occupational Health experts suggest that FCPs’ learning needs should cover the full range of return to work planning, workplace negotiation, and occupational health knowledge. Experts stress that those with positive attitudes on work topics tend to show more confidence in managing workplace issues, with research linking confidence to prior training and education.^24^

As gatekeepers in primary care, FCPs are well positioned to manage sickness absence within a biopsychosocial framework that views work as an essential part of health and rehabilitation. Nonetheless, limited data suggest that FCPs rarely provide specific work advice, with one evaluation showing only 29% of employed patients receiving work advice.^1^ National Health Service data also indicate that, despite a 10% increase in Fit Notes issued after legislative expansion, 95% still follow the ‘Not Fit for Work’ format.^30^ This suggests that a reliance on the ‘Sick Note’ model persists, with many physiotherapists viewing work outcomes as secondary to clinical responsibilities. Bridging this gap requires competencies that align work and health goals, enabling FCPs to support patients in achieving positive health and work outcomes.

Integrating a biopsychosocial approach that includes work as part of health recovery allows FCPs to better support patients in managing work related musculoskeletal conditions. Physiotherapists often act as primary providers with ongoing patient relationships, positioning them to take on work focused roles for working age adults. Consistent and right competencies in work and health would enable physiotherapists to address work related factors effectively, promoting sustainable work participation.^31^ Physiotherapists’ role in minimising the impact of musculoskeletal issues on patients’ work and life can be significant. Although practice norms vary internationally, this competency framework provides foundational guidance for adapting work focused care approaches across settings, regardless of regional legislative or practice differences.

In conclusion, establishing structured competencies in work and health for FCPs is an important step towards integrating work focused care into primary practice. This approach may ultimately enhance patient outcomes, reduce sickness absence, and address broader health and economic challenges.

**Clinical messages**

- Musculoskeletal conditions are a major contributor to years lived with a disability and early exit from the workplace.
- FCPs can become occupational health champions in primary care settings and address the impact of musculoskeletal conditions, providing a competency-based approach to work and health training and development is offered.
- Future research could further evaluate the use of Fitness For Work strategies and Sickness Absence certification within the primary care FCP model of practice and asses if the legislative changes have improved the effectiveness of the ‘Fit Note’.
